# Complementary Feeding Practices for South Asian Young Children Living in High-Income Countries: A Systematic Review

**DOI:** 10.3390/nu10111676

**Published:** 2018-11-05

**Authors:** Logan Manikam, Raghu Lingam, Isabel Lever, Emma C. Alexander, Chidi Amadi, Yasmin Milner, Taimur Shafi, Lucy Stephenson, Sonia Ahmed, Monica Lakhanpaul

**Affiliations:** 1 UCL Institute of Epidemiology & Health Care, 1-19 Torrington Place, London WC1E 6BT, UK; 2 Population Child Health Research Group, School of Women’s and Children’s Health, University of New South Wales, Sydney 2031, Australia; r.lingam@unsw.edu.au; 3 GKT School of Medical Education, Guy’s Campus, London SE1 1UL, UK; isabel.lever@kcl.ac.uk (I.L.); emma.alexander@kcl.ac.uk (E.C.A.); yasmin.milner@bartshealth.nhs.uk (Y.M.); t.t.shafi@gmail.com (T.S.); 4 Royal Surrey County Hospital, Egerton Road, Guildford GU2 7XX, UK; chidi.amadi@btinternet.com; 5 Population, Policy & Practice, UCL Great Ormond Street Institute of Child Health, 30 Guilford Street, London WC1N 1EH, UK; lucy.stephenson.10@ucl.ac.uk (L.S.); sonia.ahmed@ucl.ac.uk (S.A.); m.lakhanpaul@ucl.ac.uk (M.L.)

**Keywords:** infant, diet, child, nutrition, complementary feeding, high-income countries

## Abstract

Sub-optimal nutrition among South Asian (SA) children living in high-income countries is a significant problem. High rates of obesity have been observed in this population, and differential complementary feeding practices (CFP) have been highlighted as a key influence. Our aim was to undertake a systematic review of studies assessing CFP in children under two years of age from SA communities living in high-income countries, including dietary diversity, timing, frequency and promotors/barriers. Searches covered January 1990–July 2018 using MEDLINE, EMBASE, Global Health, Web of Science, BanglaJOL, OVID Maternity and Infant Care, CINAHL, Cochrane Library, POPLINE and World Health Organisation (WHO) Global Health Library. Eligible studies were primary research on CFP in SA children aged 0–2 years. Search terms were “children”, “feeding” and “South Asian”, and derivatives. Quality appraisal used the Evidence for Policy and Practice Information (EPPI) Weight of Evidence scoring. From 50,713 studies, 13 were extracted with ten from the UK, and one each from the USA, Canada and Singapore. Sub-optimal CFP were found in all studies. All ten studies investigating timing reported complementary feeding (CF) being commenced before six months. Promoters/barriers influencing CFP included income, lack of knowledge, and incorrect advice. This is the first systematic review to evaluate CFP in SA children living in high-income countries and these findings should inform the development of effective interventions for SA infants in these settings.

## 1. Introduction

The World Health Organisation (WHO) defines complementary feeding (CF) as “the process starting when breast milk alone is no longer sufficient to meet the nutritional requirements of infants, and therefore other foods and liquids are needed” [1]. CF therefore focuses on bridging the gradual transition from exclusive breastfeeding to solid foods eaten alongside the whole family in conjunction with breastfeeding. The WHO Infant and Young Children Feeding (IYCF) guidelines, an internationally ratified framework adopted in most high-income countries, state that infants should be exclusively breastfed for the first six months of life to achieve optimal growth, development and health [1]. Thereafter, infants should receive safe and nutritionally adequate complementary foods while breastfeeding continues up to 24 months or beyond. The IYCF guidelines, whilst recommended for high-, middle- and low-income countries, have a major focus towards middle- and low-income countries, including South Asian (SA) countries, such as India, Pakistan, and Bangladesh [2]. 

Despite clear guidelines, inappropriate complementary feeding practices (CFP) are common even amongst those with knowledge of guidelines [3], and have been linked to childhood obesity and malnutrition [4]. A 2016 meta-analysis reported that early complementary feeding <4 months is associated with an increased risk of being overweight (Relative Risk (RR) 1.18) and being obese (RR 1.33) in childhood [5]. In 2016, 18.4% of children in high-income countries were overweight, and 13.6% were obese [6]. The problem of obesity is particularly prevalent amongst children of South Asian (SA) origin living in high-income countries. In a 2011 report on childhood obesity, fewer White British children aged 4–5 years were obese (9.6%) compared to Bangladeshi (15.1%), Pakistani (11.8%), and Indian (9.7%) children, with CFP highlighted as an influencing factor [7]. Childhood obesity in these children is linked to an increased risk of early onset type 2 diabetes, a disease which South Asians are already more susceptible to [8].

In addition to obesity, inappropriate nutrition can result in dental caries, micronutrient deficiency and also low weight gain. South Asian infants in high-income countries have been reported as having a higher prevalence of caries compared to white infants [9,10]. In a 2011 report, SA infants had lower intakes of iron, sodium, thiamine, and magnesium relative to white infants, amongst other dietary differences [11].

These risks present a clear opportunity for public health interventions. When CFPs are adhered to correctly, there are reduced rates of malnutrition and better infant growth [12]. Existing interventions include the UNICEF and WHO “Global Breastfeeding Advocacy Initiative,” involving health visitors, midwives, the provision of audio tapes, written postnatal information and the bolstering intrinsic motivation amongst South Asian (SA) families [13,14,15].

We therefore aimed to review CFP in line with WHO IYCF guidelines for SA families residing in high-income countries. As part of the same project, we simultaneously reviewed CF practices for SAs in India, Pakistan, and Bangladesh, with these countries being the most populous SA countries; these three reviews are published elsewhere [16,17,18]. 

## 2. Materials and Methods 

A protocol for this review was registered on PROSPERO, Registration No: CRD42014014025.

### 2.1. Eligibility Criteria

Studies were included if they met the following criteria:

- Participants: Children aged 0–2 years of SA descent living in high-income countries according to World Bank definitions [19].
- Outcomes: Adequacy of CF (based on minimum dietary diversity and meal frequency), timing of introduction of CF and barriers/promoters to incorporating WHO-recommended CFP
- Language: Studies published in English, or with translation available
- Year: Published from 1990–2018

All study types (qualitative, quantitative or mixed) were included to ensure the diversity of evidence was captured. We excluded studies solely focusing on exclusive breastfeeding and interventional studies. 

Although various other CF guidelines are referenced in high-income countries, such as the European Society for Paediatric Gastroenterology Hepatology and Nutrition (ESPGHAN) guidelines [20], the IYCF guidelines were chosen to frame the review in order to enable consistent comparison between high- and middle- or low-income countries, and also because many high-income countries use these guidelines as a basis for their own country-specific advice [21,22,23]. 

### 2.2. WHO IYCF Definitions of Adequate Complementary Feeding

Adequacy regarding the introduction of CF is assessed on the proportion of infants aged 6–8 months who receive solid, semi-solid or soft foods. 

Minimum dietary diversity is assessed by the proportion of children 6–23 months of age who receive foods from four or more food groups. The seven WHO IYCF recommended food groups consist of [21];

(1) Grains, roots and tubers
(2) Legumes and nuts
(3) Dairy products (e.g., milk, yoghurt, cheese)
(4) Flesh foods (e.g., meat, fish, poultry, and liver/organ meats)
(5) Eggs
(6) Vitamin A rich fruits and vegetables
(7) Other fruits and vegetables

Infants who receive at least four of the seven food groups on a given day are defined as having a diet that meets the minimum recommended level of dietary diversity. 

Minimum meal frequency is assessed by the proportion of infants 6–23 months of age who receive solid, semi-solid, or soft foods according to the IYCF recommended minimum number of feeds per day. This is two times a day for breastfed infants 6–8 months old, three times a day for breastfed infants 9–23 months old, and four times a day for non-breastfed infants 6–23 months old [21]. 

### 2.3. Information Sources

A search strategy was devised to search the following databases: MEDLINE, BanglaJOL, Embase, Global Health, Web of Science, OVID Maternity and Infant Care, The Cochrane Library, POPLINE and WHO Global Health Library. Searches were originally conducted in December 2014 and updated twice, in June 2016 and July 2018. Members of electronic networks on @jiscmail.ac.uk, including minority-ethnic-health and networks (e.g., The South Asian Health Foundation) were contacted to request any additional or unpublished material. Bibliographies of included articles were hand-searched.

### 2.4. Search Strategy

The search strategy included terms for “feeding”, “South Asian” and “children”. The search strings used for MEDLINE were:

Term 1: Children < 2 years 

Infant OR Baby OR Babies OR Toddler OR Newborn OR Neonat * OR Child OR Preschool OR Nursery school OR Kid OR Pediatri * OR Minors OR Boy OR Girl

Term 2: Feeding 

Nutritional Physiological Phenomena OR Food OR Feeding behavior OR Feed OR Nutrition OR Wean OR fortif * OR Milk

Term 3: South Asian 

Ethni * OR India * OR Pakista * OR Banglades * OR Sri Lanka OR Islam OR Hinduism OR Muslim OR Indian subcontinent OR South Asia 

Use of ‘*’ indicates that the word acts as a stem.

### 2.5. Study Selection and Data Extraction

In total, 50,713 titles and abstracts were screened against inclusion criteria by two independent reviewers, with conflicts resolved by discussion. In total, 49,735 titles and abstracts were excluded. This left 978 potentially eligible full text articles, which were independently reviewed by two researchers. One hundred and thirty-nine full text articles were ultimately extracted, of which 13 were relevant to high-income countries and the remainder relevant to India, Bangladesh and Pakistan [16,17,18]. 

Data was extracted initially by a single reviewer and validated by a second using a piloted modified worksheet including: Country of study; study type; study year; study objectives; population studied, eligibility criteria and illness diagnosis; study design; ethical approval; sampling; data collection and analysis; feeding behaviours; adequacy of CFP; timing of initiation of CF; weight of evidence. Some studies utilised different classification categories from the WHO IYCF guidelines (e.g., when assessing commencement of CF, their age range classifications overlapped with the IYCF categories) and they were classified into multiple IYCF categories as appropriate. 

### 2.6. Result Synthesis

To standardise study classifications, the formal definitions below were utilised and applied:

(1) Cohort study; An observational study in which a group of patients are followed over time. These may be prospective or retrospective.
(2) Cross sectional study; An observational study that examines the relationship between health-related characteristics and other variables of interest in a defined population at one particular time.
(3) Mixed methods; A study that combines both quantitative and qualitative methodology.

In view of the considerable heterogeneity of methodology and outcomes, a narrative approach to synthesis was utilised using guidance developed from the University of York Centre for Reviews and Dissemination (CRD) and the Economic and Social Research Council (ESRC) [24,25,26,27].

The evidence reviewed is presented as a narrative report with results broadly categorised following IYFP indicators on (1) adequacy of CFP comprising of dietary diversity, meal frequency, timing of introduction of CFP, consumption of iron-rich foods and sources of advice for feeding, and (2) barriers/promoters influencing CFP.

Barriers were defined as impediments to achieving correct CF whilst promoters were defined as supporters to achieving correct CFP. These were subcategorised into factors influencing at the family (e.g., family members), and organisational level (e.g., health care providers, hospitals, political bodies).

### 2.7. Quality Assurance

The Centre for Reviews and Dissemination (CRD) guidance emphasises the importance of using a structured approach to quality assessment when assessing descriptive or qualitative studies. However, it acknowledges the lack of consensus on the definition of poor quality with some arguing that using rigid quality criteria lead to the unnecessary exclusion of papers.

In our review, the Evidence for Policy and Practice Information and Co-ordinating Centre (EPPI-Centre) Weight of Evidence Framework was used to allow objective judgements about each study’s value in answering the review question [28]. It examines three study aspects: Quality of Methodology, Relevance of Methodology and Relevance of Evidence to the Review Question and categorises them to Low, Medium or High. An average of these weightings establishes the Overall Weight of Evidence of a study. Studies with an Overall Weight of Evidence of Low were to be included in the table of included studies, but not discussed further within the results and discussion. This assessment was performed by two independent reviewers, with arbitration where required. 

## 3. Results

**Figure 1:**
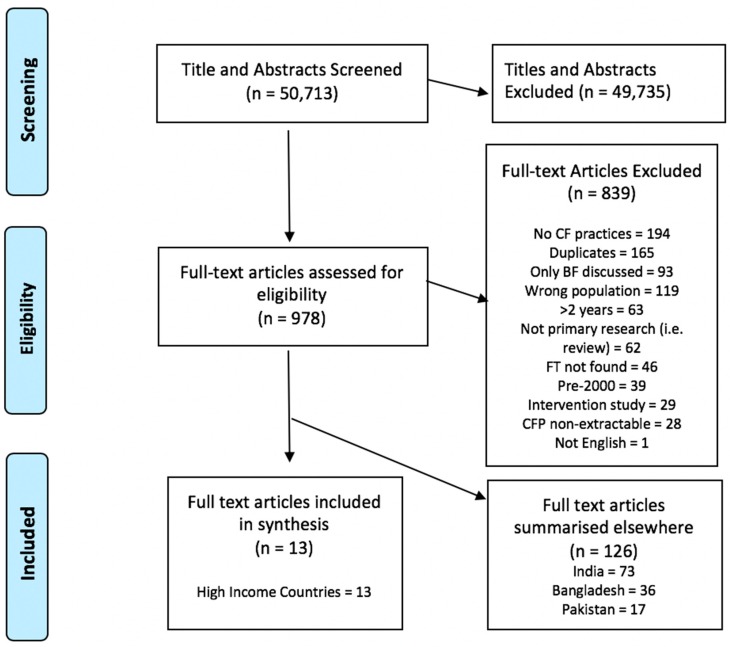
Preferred Reporting Items for Systematic Reviews and Meta-Analyses (PRISMA) flow diagram—study selection process. CF, complementary feeding; CFP, complementary feeding practices; BF, breastfeeding; FT, full text.

Of the 50,713 studies identified, 13 studies focusing on CFP in SA families in high-income countries were included in this systematic review. The study selection process is denoted in Figure 1.

### 3.1. Study and Participant Characteristics

These 13 studies consisted of eight cohort and five cross-sectional studies. Ten studies took place in the United Kingdom, and one each in the United States, Canada and Singapore. In total, the listed study populations incorporated 1559 infants, 1878 mothers or parents, 1834 families, and 320 mother-child pairs included across the 13 studies.

Table 1 presents the “Weight of Evidence” (WOE) awarded to each of the studies. Eight studies received a WOE rating of Medium, and five a WOE rating of High. Table 2 denotes a summary of the evidence across all included studies.

### 3.2. Adequacy of Complementary Feeding

As per the WHO IYCF indicators, adequacy of CFP is assessed according to minimum dietary diversity (MDD), meal frequency and timing of introducing CFP. These are detailed below with a further section discussing advice providers. 

### 3.3. Dietary Diversity

Table 3 summarises all complementary foods groups identified from the studies categorised according to the WHO IYCF food groups. Overall, nine of the 13 studies reported food or types of food used for CF. Minimum dietary diversity is assessed by the proportion of children 6–23 months of age who receive foods from four or more food groups; formal dietary diversity using this definition of minimum dietary diversity was not calculated by any study. 

The most popularly reported complementary food groups were “fruit and vegetables”, “flesh foods”, and “dairy products”, which were each identified by six of nine studies. Five studies reported “grains, roots and tubers” being used for CF, three studies “eggs”, and two studies “legumes and nuts”. None of the studies specifically identified or investigated intake of “Vitamin A-rich Fruit and Vegetables”, despite this being one of the IYCF categories. 

Fruit and vegetables were mentioned as part of CF in six of nine studies. Thomas and Avery (WOE = H) found that at nine months of age, fruits were given at least once a day to 32% of Bangladeshi infants, 50% of Pakistani infants, and 48% of Indian infants in their UK-based cohort [39]. Vegetables were provided daily for 25% of Bangladeshi infants, 13% of Pakistani infants, and 33% of Indian infants. Toh et al. (WOE = H) reported that fruit puree was the first food for 11.6% of the Indian-origin infants in their Singaporean cohort, with vegetable puree being the first food for 5.8% [40]. In Sarwar (WOE = H) 12% of Pakistani mothers living in England provided fruit as a first food, and 7% used vegetables [37].

Flesh foods were also described as part of CF in six of nine studies. Several studies specified ‘meat’ as being part of CF [30,33,37,39] or processed meat [35]. Others described fish as being given [30,39,41]. Thomas and Avery (WOE = High (H)) reported that at nine months of age, meat was eaten at least once a day by 8% of Bangladeshi children, 10% of Pakistani children, and 19% of Indian children, compared to 50% of White children [39]. Kannan et al. (WOE = Medium(M)) reported that the most frequent first meat fed to Asian-Indian American infants was chicken with vegetables [33]. However, Sahota et al. (WOE = H) found that British Pakistani infants were less likely to consume processed meat products compared to White British counterparts at both 12 and 18 months of age [35]. 

Four studies highlighted the addition of high sugar products as part of CF. Santorelli et al. (WOE = H) in the “Born in Bradford” study found a higher proportion of Pakistani (59.8%) and other SA mothers (58.2%) utilising unrefined sweetened foods as a first food compared to 36.9% of White British mothers [36]. Sarwar (WOE = H) found that Pakistani mothers who lived in England were more likely to offer sweet convenience food when initiating CF compared to mothers living in Pakistan (40% vs. 2%) and in a UK study Dykes et al. (WOE = M) reported that 19.8% of Bangladeshi mothers, 10.7% of Pakistani mothers and 6.6% of Indian mothers added sugary foods, such as sugar, honey and biscuits to the bottle at nine months, items that do not fit into any IYCF category [31,37]. Sahota et al. (WOE = H) also observed that, at 12 months, British Pakistani infants were more likely to consume sugar-sweetened drinks (adjusted odds ratio 1.68) and commercial sweet baby meals (adjusted odds ratio 1.90) than White British infants [35]. Separately, Toh et al. (WOE = H) reported that Indian infants living in Singapore were significantly (*p* < 0.001) more likely to have oil added to their foods than Chinese and Malay infants at both 9 and 12 months of age [40].

### 3.4. Frequency and Timing 

#### 3.4.1. Meal Frequency

Minimum meal frequency is assessed by the proportion of infants 6–23 months of age who receive solid, semi-solid, or soft foods according to the IYCF recommended minimum number of feeds per day [21]. Meal frequency was analysed by Thomas and Avery (WOE = H), but not in the other 12 studies [39]. Thomas and Avery assessed the practices of South Asian mothers living in England, and found that for Bangladeshi, Pakistani and Indian families, the proportion of infants consuming three meals a day steadily increased in proportion with age. Indian families were the most likely to maintain three meals a day beyond the age of six months with 75% (Indian families) doing so compared with 59% (Bangladeshi families) and 61% (Pakistani families). These numbers increased at nine months to 93% compared to 83% and 85%, and at 12 months to 99% compared to 97% and 98% [39]. By 15 months, they reported that 100% of babies consumed three meals a day across all nationalities. 

#### 3.4.2. Timing of Introducing Complementary Feeding

Ten studies investigated timing of introduction of CF. Adequacy regarding the timed introduction of CF is assessed on the proportion of infants aged 6–8 months who are commenced on solid, semi-solid or soft foods. We found that CF was commenced at less than six months in all ten studies [29,30,33,34,36,37,38,39,40,41], and also at 6–8 months in five studies [34,37,38,39,40]. No studies reported any more than tiny minorities of CF commencing at later ages (from nine months up to two years). An alternative classification of timing in line with the ESPGHAN guidelines is available as Supplementary Table S1 [20].

Some of the studies reporting CF commencing before six months indicated this practice was highly predominant. Extremely early weaning was reported in Thomas and Avery (WOE = H) who noted that approximately 70% of SA families had begun weaning by three months, with this number rising to 98–99% by six months [39]. In Stearns et al. (WOE = M) 88.33% of sampled SA infants living in Canada commenced feeding with solids at 3–6 months of age, followed by 9.44% at 6–9 months, and 1.11% each at 0–3 months or 9–12 months [38]. Moore et al. (WOE = M) found that 100% of their sample of South Asian parents commenced weaning by 29 weeks (approximately 6.7 months), and the mean weaning age was 23.0 weeks [34]. Toh et al. (WOE = H) found that 97.1% of Indian infants had received their first foods before 32 weeks (7.4 months) [40].

Sarwar (WOE = H) compared practices amongst Pakistani mothers in England against Pakistani mothers in Pakistan [37]. They found that the proportion of mothers commencing weaning between the age of three and six months was not significantly different between families in Pakistan and those living in England (40% vs. 49%, respectively), however, there were a higher proportion of mothers not weaning until the age of seven months or later in Pakistan (26%), compared to in England (15%) [37]. 

There were mixed results when comparing practices of the South Asian population with white and Anglo-American populations. Santorelli et al. (WOE = H) found that Pakistani mothers (20.6%) and other SA mothers (12.1%) were less likely to introduce solid foods early at <4 months compared to White British mothers (37.1%) [36]. Similarly, Griffiths et al. (WOE = H) found that Indian, Pakistani and Bangladeshi mothers were less likely to introduce solids at <4 months than white mothers, with 17%, 12% and 14% doing so compared to 37% of white mothers [32]. Conversely, Kannan et al. (WOE = M), who compared Anglo-American and Asian-Indian American mothers, found that Anglo-American mothers tended to start CF later [33].

### 3.5. Sources of Advice

Seven studies included detailed information about advice providers and decision makers. All seven [29,33,34,37,39,40,41] studies reported that parents received advice from family, with five [33,34,37,40,41] particularly specifying the role of the parent’s mother/mother-in-law. Five studies also specified that SA parents received advice from health professionals [29,33,34,39,41].

Sarwar (WOE = H) found that the majority of mothers, both in Pakistan and Pakistani mothers living in England, received general advice on weaning from family and friends, whose advice sometimes differed from the evidence-based advice of professionals [37]. Kannan et al. (WOE = M) observed that Asian-Indian American mothers identified maternal/paternal grandmothers as the main advisor for the first six months, but made use of health professionals’ advice afterwards [33]. In Singapore, Toh et al. found grandparents were the main food decision maker for 13.0% of Indian infants, compared to 19.8% of Chinese infants and 12.9% of Malay infants [40].

In Williams and Sahota (WOE = M), mothers reported that mothers-in-law and extended family encouraged the use of sweetened or fruit drinks as alternatives to milk [41]. Other sources of information self-reported by mothers in this study included midwives, health visitors and television [41]. Moore et al. (WOE = M) found that health visitors were the most influential advice source for 31% of SA mothers, slightly below the mother/grandmother (for 32%) [34]. Thomas and Avery (WOE = H) also listed health professionals, family and media as sources of advice, and Condon et al. (WOE = M) highlighted family members, health professionals, religious texts and exposure to ‘British custom’ [29,39].

### 3.6. Barriers and Promoters 

#### 3.6.1. Factors Associated with CFP

Table 4 summarises the factors influencing appropriate CFP. Five promoters and four barriers to appropriate CFP were identified. 

#### 3.6.2. Promoters

Two studies identified promoters at a family level; these were education and understanding of guidelines [31,34]. Three studies identified promoters at an organisational level; these were healthcare professionals, and the provision of booklets of information [34,37,41]. 

##### Education

One study noted the positive influence of education. Dykes et al. (WOE = M) found that there was a significant difference in the extent to which bottles were supplemented with inappropriate sugar/sugary foods amongst Pakistani mothers, depending on education status [31]. Only 5.3% of those who stayed in education until at least 18 years supplemented inappropriately, compared to 11.8% of those who left before 18 years. However, there was no significant difference depending on education status amongst Bangladeshi or Indian mothers. 

##### Understanding of Guidelines

One study emphasised understanding of guidelines as an important promoter. Moore et al. (WOE = M) found that having accurate understanding of weaning guidelines resulted in a statistically significant later weaning, independent of demographic factors and sources of advice (*p* < 0.001) [34]. Of their sample, 66% of SA parents had an accurate understanding of the UK Department of Health weaning guidelines to wean at or around six months.

##### Healthcare Professionals 

Three studies noted that healthcare professionals promoted appropriate CFP. Sarwar (WOE = H) identified that for Pakistani mothers in Nottingham, UK, health visitors and nurses conducting home visits were the most influential healthcare professionals to give information [37]. Healthcare professionals gave information to 95% of these mothers about when to introduce solids to the baby, 89% about which solids should be given, and 62% about how often to feed the baby. Mothers felt that demonstrations and group discussions organised by healthcare professionals at clinics were useful. Williams and Sahota (WOE = M) found that most of the first-generation Muslim Asian mothers in their study would attempt to comply with a doctor’s advice where possible [41]. Moore et al. (WOE = M) found that 85% of health visitors advised parents to commence weaning at or around six months, compared to 46% of family members, and receiving weaning advice from a health visitor was associated with a statistically significant later weaning age (*p* < 0.001) [34].

##### Provision of Information 

One study noted the provision of information as important. Sarwar (WOE = H) noted that some Pakistani mothers in Nottingham were given booklets written in both English and Urdu [37]. Sources of information, such as booklets and audio tapes were seen as desirable, preferably in the mother’s traditional language. 

#### 3.6.3. Barriers 

Four studies identified barriers at a family level; these were familial pressure, incorrect knowledge, and low income [31,37,39,41]. Three studies identified a barrier at the organisational level; the barrier posed by bad interactions or incorrect advice from healthcare professionals [29,37,41].

##### Familial Pressure

Two studied noted familial influence as a barrier. Mothers were reported as receiving much of their information about CFP from family members and feeling obliged to follow it. Sarwar (WOE = H) found that most mothers were given advice by the family. This often conflicted with information given by healthcare professionals, causing confusion, however some mothers still felt pressured to follow family advice over nutritional education they had received [37]. Williams and Sahota (WOE = M) also noted that some mothers felt pressure from mothers-in-law and therefore felt they had a lack of control over dietary issues [41].

##### Knowledge 

Incorrect knowledge about correct CFP was a barrier reported by two studies. Both studies were conducted in the 1990s (1990 for Williams and Sahota, and 1997 for Thomas and Avery) [39,41]. Williams and Sahota (WOE = M) found that drinks, such as Ribena and orange juice, were being given at three months by some mothers because they believed it was healthy, would provide vitamins, give strong bones and relieve constipation [41]. Thomas and Avery (WOE = H) found that mothers were giving drinks other than milk at nine weeks, because they believed the child was thirsty or dehydrated, it was good for the child, or that it relieved constipation, colic, wind and hiccups [39]. 

##### Income

One study reported the barrier posed by a low income. Dykes et al. (WOE = M) completed regression models of the effect of various factors on whether sugary foods would be added to the bottles of infants [31]. They found that the poorest families were most likely to supplement with inappropriate sugary foods, with the first quintile of equivalised income having an odds ratio over the fifth quintile of between 5.14–4.86 across each of three models.

##### Interactions and Advice from Healthcare Professionals

Three studies reported the conduct of healthcare professionals as sometimes acting as a barrier. Williams and Sahota (WOE = M) reported in 1990 of instances where healthcare professionals were a barrier to good CFP [41]. Within their study, three discussion groups reported that they gave drinks other than milk at three months because it was “recommended by the health visitor”. Some mothers described health visitors and midwives giving contradictory information. There was also a perceived lack of post-natal support, with two groups describing nursing staff laughing at them when they were unable to breastfeed properly. Condon et al. (WOE = M) described in 2003 that the common practice was for weaning to begin at four months, as it was believed this was in accordance with health professional advice at the time [29].

Sarwar (WOE = H) described that all the mothers in their study expressed a desire for more information from healthcare professionals [37]. Some of the mothers lacked confidence in the advice of healthcare professionals, and some mothers had difficulty communicating due to linguistic barriers.

## 4. Discussion

To our knowledge, this is the first systematic review to assess CFP in SA families living in high-income countries. In many SA families in these settings, WHO IYCF standards on minimum dietary diversity and timing of introducing CF were not being met. 

### 4.1. Comparison with SA Countries of Origin

Other papers published by our group as part of this project reviewed CF practices in Bangladesh, Pakistan, and India [16,17,18]. As reported in this review, the most common CF food groups for SA infants in high-income countries were “fruits and vegetables”, “flesh foods” and “dairy products”, described by six studies each, whereas the other reviews found “Grains, roots and tubers” were most commonly used for CF in the SA countries themselves. For example, “Grains, roots and tubers” were reported by 26 of 27 studies investigating dietary diversity in Bangladesh [17].

Complementary feeding was reported as occurring predominantly before six months in all ten studies that investigated timing; conversely, in Pakistan, Bangladesh and India, six to nine months was the most common time period for the commencement of CF for each country [16,17,18]. Although meal frequency was measured by only one study in this review, it was encouraging that by 15 months, 100% of babies consumed three meals a day across all nationalities [39]. The proportion of infants meeting minimum meal frequency (MMF) from SA countries in the concurrent reviews was variable and MMF rates of 25% or below were common. Factors that could influence this finding may include income or differing health service provisions. 

Most of the barriers to appropriate CF reported in this review were common to those reported in SA countries. The barriers at the family level of familial pressure, low income and incorrect knowledge were also mentioned in Pakistan, Bangladesh and India [16,17,18]. The promoter statuses of education, good advice and good advice sources, especially from healthcare professionals, were likewise shared by many of the studies included across all three countries. 

### 4.2. Comparison with Non-SA Residents of High-Income Countries

Ten of the thirteen papers reviewed focused on South Asian families in the UK with little on other high-income countries, with one study each set in the USA, Canada and Singapore. In recent census surveys, South Asians made up 4.9% of UK population, 1.2% of the population in the USA, 4.8% of the population in Canada and at least 9% of the population in Singapore [42,43,44,45]. This limits the generalisability of our findings to South Asian communities in other high-income countries, such as Australia, where the SA population is at least 4% [46], but also highlights that continuing research in these settings is important. 

Studies in high-income countries have found that commencing CF before six months is also relatively common. The largest recent study of CF across the UK was the 2010 Infant Feeding Survey [47]. A total of 10,768 mothers provided full questionnaire responses. The introduction of solids before six months was commonplace, with 94% having introduced solids by six months, and 75% by five months of age, even though the UK Department of Health has advised exclusive breastfeeding for six months since at least 2003 [48]. It was also reported that 59% of infants aged seven months or above receiving solid foods were having at least three meals a day. The most common first food provided to babies was baby rice, for 57%, followed by 12% using ready-made baby food [47]. A study of 1482 infants in the US, making use of data from the 2009–2014 National Health and Nutrition Examination Survey, found that later introduction of CF was slightly more prevalent; only 39% had been introduced to CF by five months, compared to 55% by six months and 87% by seven months of age [49].

### 4.3. Implication of Key Findings

Few studies reported CF involving legumes and nuts or eggs, particularly when compared to practices in India, Pakistan and Bangladesh. There was a reduced intake of meat by SA infants relative to white infants, which may well be influenced by cultural and religious restrictions that promote vegetarianism or veganism and restrict meats, such as pork. Vegetarianism is much more prevalent amongst South Asians than in most high-income comparison countries, with one study finding a prevalence of 33% amongst South Asian adults compared to 2.4% of adults in the US [50]. Interestingly, iron intake amongst Asian Indian American infants exceeded recommended dietary allowances [33,51]. 

No study strictly followed the IYCF categorisation of food groups as part of the analysis, and thus it was not possible to assess intake of Vitamin-A rich fruit and vegetables. It will be important to accurately characterise the extent to which CF is limited in diversity for this category in future studies, because severe Vitamin A deficiency can weaken the immune system and cause anaemia and night blindness [52]. Vitamin A deficiency amongst preschool children has been categorised as severe (>20% prevalence) in India and Bangladesh themselves, and Moderate (10–20%) in Pakistan, but is much less common in high-income countries [53,54].

This review found that most studies reported SA infants in high-income countries beginning weaning early (before six months), compared to the ideal time of between 6–8 months as recommended by the WHO IYCF guidelines; later weaning was more common in SA countries themselves. Whilst IYCF guidelines are applicable to both high and middle-to-low income countries, they focus on the latter [2]. However, many high-income countries also adhere to the WHO guidelines and use them as a basis for developing their own; the UK Department of Health guidelines have recommended since 2003 and in their most recent publications that infants should be offered solid foods “from six months” with similar food groups recommended as in the WHO IYCF guidelines [23,48,55,56]. In Canada, exclusive breastfeeding is recommended for the first six months of life, and the American Academy of Pediatrics recommend the same; in Australia, exclusive breastfeeding is recommended until “around” six months [22,57,58]. However, the European Society for Paediatric Gastroenterology, Hepatology and Nutrition (ESPGHAN) committee recommends that “complementary feeding (…) should not be introduced before 17 (4 months) weeks and not later than 26 weeks (end of the 6th month)”, although “exclusive or predominant breastfeeding for approximately six months is considered a desirable goal” [20]. The influence of other guidelines on advice delivered by health professionals may be linked to the finding of this study that CF commencing before six months is commonplace amongst SA infants in high-income countries, alongside the influence of since-revised guidelines in earlier-published studies. Alongside greater penetration of WHO IYCF guidelines in South Asia, it has been suggested that later weaning in SA countries may be related to perceptions that the water supply is less safe, and breast milk should be relied upon [59]. This may be a concern that is less common for families residing in high-income countries.

It will be important to complete large-scale, prospective studies that can accurately characterise the link between inadequate complementary feeding and the development of obesity. Systematic reviews and meta-analyses of timing have found associations between very early (<4 months) CF and being overweight or obese in childhood [5,60]. and a UK-based study found an association between guideline adherence regarding dietary variety and having a higher lean mass [61], but future studies should examine the association between obesity and inadequate CF in other domains. The lack of such studies is a limitation in the field. 

Data showing that South Asian mothers are more likely to offer sweetened foods at initiation of CF is especially relevant given the high rates of obesity and dental caries amongst South Asian children [7,9,10,36,37]. Appropriate CF is important both in preventing undernutrition and being overweight [62]. A review of the evidence of interventions to support appropriate CFP in minority groups identified two studies targeting South Asian families in the UK [63]. One made use of dietary education delivered by a link worker and reported improved nutritional practices; a second also made use of link workers conducting home visits to offer advice, and reported that at one year 92% of families exposed to the intervention were providing a varied diet [64,65]. Such interventions should be considered for further development in high-income countries to optimise nutritional outcomes for this at-risk group. 

Finally, an important influencing factor for appropriate CFP is the provision of information. Some of the earlier studies reported in this review particularly mentioned the influencing power of information and incorrect advice from HCPs; it is hoped that the greater permeation of the WHO IYCF and other guidelines may mean the latter is less of a problem for current families [20,21]. The influence of information supports the development of, in particular, educational interventions to foster appropriate CFP [66,67]. Especially for first-generation immigrants, exposure to advice from conflicting guidelines may cause uncertainty, especially given the identified role of family members as a further source of advice. More effort should be put into targeted interventions for at-risk individuals, especially those from low income backgrounds [68]. The provision of audio tapes in women’s traditional language and demonstrations or group discussions organised by healthcare professionals could be easy interventions for local centres to implement themselves [37]. 

### 4.4. Strengths and Limitations

The strengths of our systematic review are derived from searching a large number of databases utilising broad search strings, performing updated searches in 2016 and 2018, and having two reviewers undertake study selection, data extraction and quality assessment.

Key limitations included the exclusion of papers that solely focused on children over two years where CFP described in their younger years may have been missed, papers published before the year 1990 at full text review and papers not published in English. 

The inclusion of studies from the 1990s and early 2000s in this review means that conclusions about adequacy of CFP in this population do not fully reflect current practice, instead reflecting recent practice, and the influence of different guidelines over time may have affected findings. Four studies out of 13 in this review were published in the 1990s [30,33,39,41].

Additionally, we did not assess the quantities of the foods used, only the frequency with which they appeared in the studies. The eligible studies tended to address broad research questions, were conducted using qualitative and/or quantitative and/or descriptive methods and were not presented following standardised reporting guidelines (e.g., STROBE or COREQ). Meta-analyses were therefore not undertaken. Finally, whilst we attempted to contact numerous authors to identify relevant grey literature for our review, due to the breadth and depth of the field of nutritional research, this is unlikely to be exhaustive with publication bias likely to be present. 

## 5. Conclusions

This is the first systematic review to assess CFP amongst the SA population living in high-income countries. The study has found that there is a relative dearth of evidence on CFP in this setting compared to SA countries of origin. It was also found that dietary diversity was variable, and timing of introduction of CF was commonly occurring before six months, which is early according to WHO IYCF guidelines. Factors influencing appropriate CFP were similar in high-income countries compared to low- and middle-income countries, particularly relating to income and knowledge. This study should inform future researchers about the state of evidence for CFP in this setting and encourage the development of interventions to improve CFP in these communities.

## Figures and Tables

**Table 1:** Weight of evidence awarded to each study.

Author	Weight of Evidence A	Weight of Evidence B	Weight of Evidence C	Weight of Evidence D
	Quality of Methodology: The Accuracy, Coherency and Transparency of Evidence.	Relevance of Methodology: The Appropriateness of the Methodology for Answering the Review Question.	Relevance of Evidence to the Review Question: The Relevance of the Focus of the Evidence for Answering the Review Question.	Overall Weight of Evidence: Overall Assessment of the Extent to which the Study Provides Evidence to Answer the Review Question.
Condon et al. (2003) [29]	High	Medium	Medium	Medium
Duggan et al. (1992) [30]	High	Medium	Medium	Medium
Dykes et al. (2002) [31]	High	Medium	Low	Medium
Griffiths et al. (2007) [32]	High	High	Medium	High
Kannan et al. (1999) [33]	Medium	Medium	High	Medium
Moore et al. (2013) [34]	High	Medium	Medium	Medium
Sahota et al. (2015) [35]	High	High	High	High
Santorelli et al. (2014) [36]	Medium	Medium	Medium	Medium
Sarwar et al. (2002) [37]	High	High	High	High
Stearns et al. (2017) [38]	High	Medium	Medium	Medium
Thomas and Avery (1997) [39]	Medium	High	High	High
Toh et al. (2016) [40]	High	High	High	High
Williams and Sahota (1990) [41]	Medium	Low	Medium	Medium

**Table 2:** Summary of included studies.

Author	Study Title	Study Type	Location	Population	Sample Size	Diversity	Timing	Frequency	Advice	Factors
**Studies from the United Kingdom**										
Condon et al. (2003) [29]	Cultural influences on breastfeeding and weaning	Cross-sectional	Bristol, UK	Natural mothers, singleton birth, who had breastfed in the last year from Pakistani, Bangladeshi, Somali and Afro-Caribbean background.	75 (26 in focus group (17 SA), 49 in phone survey (13 SA)	Egg custard and tinned baby food were mentioned by a Bangladeshi focus group.	At 12 weeks complementary feeding (CF) had been commenced by 29% of black and 7% white mothers, 0% of Asians. By 16 weeks; 43% of Asians, 89% black and 90% white mothers.	/	Healthcare professionals, family members, religious texts, and British custom were mentioned.	Weaning began at four months as it was believed this was in accordance with healthcare professional (HCP) advice.
Duggan et al. (1992) [30]	The weaning diet of healthy Asian children living in Sheffield. 1. The level and composition of the diet in children from 4 to 40 months of age	Cross-sectional	Sheffield, UK	Healthy Asian weanlings aged 4–40 months and living in Sheffield	120 Asian children (72% born in Pakistan, 18% in Bangladesh, 10% in Britain)	Meat, fish, commercial baby foods, and fruit juices were described as being amongst foods for CF.	74% commenced weaning before six months of age.	/	/	/
Dykes et al. (2002) [31]	Socio-economic and ethnic influences on infant feeding practices related to oral health	Cohort	UK	Families with babies of Bangladeshi, Indian, Pakistani or White origin. Secondary analysis of Thomas and Avery [39]	2382 families (764 Indian, 593 Pakistani, 477 Bangladeshi, 548 White)	Described addition of sugar and sugary foods to the bottle at nine months, including sugar, honey, rusks, chocolate powder, and biscuits; 19.8% of Bangladeshi mothers did so, compared to 10.7% (Pakistani), 6.6% (Indian), 6.9% (White).	/	/	/	Education–Pakistani mothers who were in education up to 18 years were significantly less likely to supplement drinks with sugary foods (11.8% vs. 5.3%, *p* < 0.05 in those who left <18). Low income families were more likely to supplement with sugary foods.
Griffiths et al. (2007) [32]	Do early infant feeding practices vary by maternal ethnic group?	Cohort	England, UK	Natural mothers of singleton infants across England	18,150 (11,286 in England, of which 452 Indian, 857 Pakistani, 249 Bangladeshi)	/	Indian, Pakistani and Bangladeshi mothers were less likely to introduce solids early at <4 months (17%, 12%, 14% respectively) compared to 37% of white mothers. Figure 1 illustrates that close to 100% of mothers had introduced solids by six months.	/	/	/
Moore et al. (2013) [34]	Influence of weaning timing advice and associated weaning behaviours in a survey of black and minority ethnic groups in the UK	Cross-sectional	London, UK	Parents/carers recruited from London boroughs with a high percentage BME population	349 (120 South Asian, 107 Black African, 54 Black Caribbean, 64 Black mixed-race)	/	100% of SA parents had commenced weaning at 29 weeks, 87% by 26 weeks, 37% by 22 weeks. The mean weaning age for SAs was 23 weeks. The mean for Black Caribbean was 21.1 weeks and was 20.9 for Black African.	/	76% received advice from a health visitor. Other sources named as the most influential source included mother/grandmother, the internet, GP, friends, internet, and books.	Health visitor advice was associated with a later weaning age. Having a good understanding of the Department of Health weaning guidelines was associated with later weaning (*p* < 0.001).
Sahota et al. (2015) [35]	Ethnic differences in dietary intake at age 12 and 18 months: the Born in Bradford 1000 Study	Cohort	Bradford, UK	Children aged 12–18 months	1259 (473 White British, 613 Pakistani, 89 Other South Asian, 84 Other)	At 12 months, Pakistani infants consumed more commercial sweet baby meals per week (Odds Ratio (OR) 1.90), more chips/roast potatoes (OR 2.79), more sugar-sweetened drinks (OR 1.68), more fruit (OR 2.20), more pure fruit juice (OR 1.87), less processed meat products (OR 0.11), less commercial savoury baby meals (OR 0.59) than White British infants.	/	/	/	/
Santorelli et al. (2014) [36]	Ethnic differences in infant feeding practices and their relationship with BMI at 3 years of age –results from the born in Bradford birth cohort study	Cohort	Bradford, UK	Children from birth to three years of age	1326 (507 White British, 646 Pakistani, 91 Other SA, 82 Other)	Various food groups were assessed; including non-sweetened solid foods, sweetened solid foods, sweetened and non-sweetened drinks. Sweetened foods were more frequently used as first CF by Pakistani mothers (RR 1.17) compared to White British mothers.	Pakistani (RR 0.88) and Other South Asian mothers (RR 0.82) were less likely to start CF early (<17 weeks) than White British groups; 21% of Pakistani mothers did so compared to White British (37%). Pakistani and Other South Asian mothers commenced CF at a mean of 20–22 weeks.	/	/	/
Sarwar (2002) [37]	Infant feeding practices of Pakistani mothers in England and Pakistan	Cross-sectional	Nottingham (UK) and Mian Channu (Pakistan)	Mothers of weaning aged children aged 3–12 months	90 (45 in England and 45 in Pakistan)	Pakistani mothers in England most commonly use rice as a first food (55%), followed by sweet convenience food (40%), cereal (33%), eggs (26%), savoury convenience (19%), fruit (12%), vegetables and meat (7% each). At the time of the study sweet convenience food and vegetables (45% each) were most commonly eaten.	40% of Pakistani mothers vs. 49% UK mothers commenced weaning between three and four months; 26% of mothers in Pakistani and 15% in UK did not start to wean until after seven months.	/	Family and friends, in-laws, health professionals.	Familial pressure was present with sometimes conflicting advice, some mothers had lack of confidence in advice given by HCPs. Mothers were given booklets in English and Urdu in Nottingham and audio tapes were desired.
Thomas and Avery (1997) [39]	Infant feeding in Asian families	Cohort	England, UK	Families with babies of Bangladeshi, Indian, Pakistani or White origin.	2382 families (764 Indian, 593 Pakistani, 477 Bangladeshi, 548 White)	Foods used for CF were described from all groups although Vitamin A-rich fruits and vegetables were not separately investigated. Foods used included Rusk, rice cereal, bread, pasta, rice, meat dishes, vegetables, egg or dairy, fresh fruit, desserts, sweets, chocolate, beef, poultry, fish, vegetables, potatoes, yoghurt. The most common food on day before nine months interview for each group was dessert (50% Bangladeshi), fruit (48% Pakistani), non-rice cereal (63% Indian), non-rice cereal (82% white).	At nine months, 100% of Bangladeshi/Pakistani/Indian mothers had introduced CF. At six weeks, it was 1% for all groups; at three months, it was 72%, 73%, and 70% respectively; at six months, 99%, 98%, and 99% respectively.	Among Bangladeshi, Pakistani and Indian mothers, at three months, 8%, 7%, and 6% were giving three meals a day; at six months, 59%, 61%, and 75%; at nine months, 83%, 85%, and 93%; at 12 months, 97%, 98%, and 99%; at 15 months, 100%.	Listed advice providers included baby food company, health clinic, health visitor, hospital, doctor’s surgery, family and friends, mother in law, books/magazines/leaflets, public services.	The survey examined beliefs on CF and lack of knowledge was evident in a high proportion of cases.
Williams and Sahota (1990) [41]	An enquiry into the attitudes of Muslim Asian mothers regarding infant feeding practices and dental health	Cross-sectional	Leeds, UK	First generation Muslim Asian mothers; Half of the mothers originated from the Sylhet Region of Bangladesh, and half from Mirpur in Pakistan.	100 Muslim Asian mothers (50 Bangladeshi origin, 50 Pakistani origin)	Listed drinks at three months included Ribena, orange juice, water, juices, delrosa (rosehip syrup), Gripe water. Energy sources considered suitable included ‘fish, meat, eggs, soup, vegetables, butter, honey’ Apples and oranges were most frequently mentioned daily fruits.	By three months, various non-milk drinks were being given; 15% described giving extra sweeteners, such as honey and rusks.		Midwives, health visitors, television, friends, mother in law, extended family.	Barriers included inadequate knowledge and incorrect advice from a health visitor; a promoter was advice from a doctor.
**Studies from the United States**										
Kannan et al. (1999) [33]	Infant feeding practices of Anglo American and Asian Indian American mothers	Cohort	USA	Mothers of Anglo-American and Asian-Indian American children	50 mothers (25 Anglo-American, 25 Asian-Indian American)	Foods included cereal, juice, fruits, vegetables, meat; rice and banana kheer, potato podimas, dhal laddu, rice khitchri, idli, chappati, bengal gram sundal. Asian-Indian mothers most frequently used iron-fortified rice cereal in the first six months.	Mean age of introduction by Asian-Indian American vs. Anglo-American mothers of various foods: Cereal (3.1 vs. 4.2 months), fruit (3.1 vs. 4.6), juice (2.3 vs. 3.6), vegetables (3.6 vs. 5.3), meat (6.3 vs. 7.2).		Sources were family network, HCPs, paediatricians, literature, and grandmothers. For Asian-Indian mothers grandmothers were primary source for first six months.	
**Studies from Canada**										
Stearns et al. (2017) [38]	Ethnic and diet-related differences in the healthy infant microbiome	Cohort	Brampton and Peel region of Ontario, Canada	Mother-child pairs from the South Asian Birth Cohort (START-Canada).	182 South Asian and 173 White Caucasian mother-child pairs		88.33% of SA infants commenced feeding with solids at 3–6 months of age, followed by 9.44% at 6–9 months, and 1.11% each at 0–3 months or 9–12 months.			
**Studies from Singapore**										
Toh et al. (2016) [40]	Infant Feeding Practices in a Multi-Ethnic Asian Cohort: The GUSTO Study	Cohort	Singapore	Indian-origin infant-mother dyads recruited from the Singapore National University Hospital and KK Women’s and Children’s Hospital.	842 mother-infant dyads (510 Chinese, 194 Malay, 138 Indian)	For Indian infants, first food was most commonly rice cereal (42.0%) followed by non-rice cereal (16.7%), rice porridge (13.0%), fruit puree (11.6%), vegetable puree (5.8%), rice (4.3%), baby biscuit (2.2%), others (3.6%), not answered (0.7%). Indian infants were significantly (*p* < 0.001) more likely to have oil added to their foods than Chinese and Malay counterparts at 9 and 12 months of age.	The majority of Indian infants received their first foods at 24–31 weeks (58.7%) followed by 16–23 weeks (34.1%), ≤15 weeks (4.3%), and ≥32 weeks (2.9%). The time of 24–31 weeks was also most common for Chinese infants (63.7%) and Malay infants (46.4%).		Main food decision maker was mother (78.3%), grandparent (13.0%). Others (8.7%) comprised of father, secondary caregiver, shared responsibility, and not reported.	

**Table 3:** Foods utilised for CF categorised into World Health Organisation (WHO) food groups.

Food Type	Study Reference
Grains, roots and tubers	5 studies-[33,35,37,39,40]
Legumes and nuts	2 studies-[33,39]
Flesh foods (e.g., meat, fish, poultry and liver/organ meats)	6 studies-[30,33,35,37,39,41]
Dairy products (e.g., milk, yogurt, cheese)	6 studies-[29,33,35,37,39,41]
Eggs	3 studies-[37,39,41]
Vitamin A-rich fruit and vegetables	Not specified
Other fruit and vegetables	6 studies-[33,35,37,39,40,41]

**Table 4:** Factors influencing CF practices.

**Family Level**			
**Promoters**	**Study Reference**	**Barriers**	**Study Reference**
Education	1 study-[31]	Familial pressure	2 studies-[37,41]
Understanding of guidelines	1 study-[34]	Incorrect knowledge	2 studies-[39,41]
		Low income	1 study-[31]
**Organisational Level**			
**Promoters**	**Study Reference**	**Barriers**	**Study Reference**
Healthcare professionals	3 studies-[34,37,41]	Interactions and advice from healthcare professionals	3 studies-[29,37,41]
Provision of information	1 study-[37]		

## Supplementary Materials

The following are available online at http://www.mdpi.com/2072-6643/10/11/1676/s1, Table S1: Timing of initiating CF according to ESPGHAN classifications [20].
