# Supplementary material for: Complementary Feeding Practices for South Asian Young Children Living in High-Income Countries: A Systematic Review
Source: Nutrients. 2018 Nov 5;10(11):1676. doi: 10.3390/nu10111676 (PMC6266308; doi:10.3390/nu10111676)
Supplement: Supplementary file 1 [file nutrients-10-01676-s001.pdf]

Supplementary Table S1. Timing of initiating complementary feeding (CF) according to ESPGHAN classifications [20].

Infant Age	Study Proportions Within This Period [Reference]
Early (< 4 month/<17 weeks)	43% [29]<br>17% (Indian), 12% (Pakistani), 14% (Bangladeshi) [32]<br>16% [34]<br>20.6% (Pakistani), 12.1% (Other SA) [36]<br>49% [37]<br>70% (Indian), 73% (Pakistani), 72% (Bangladeshi) [39]<br>Various discussion groups reported supplementing at 3 months [41]<br>Mean introduction of 2.3 months (juice), 3.1 months (cereal) [33]<br>1.11% [38]<br>4.3% ≤15 weeks, 34.1% 16-23 weeks [40]
Timely (17-26 weeks/4-6 months)	57% [29]<br>74% commenced <6 months (detail not provided) [30]<br>Most mothers introduced solids in this period [32]<br>71% [34]<br>Mean of 20 (Pakistani), 22 weeks (Other SA) [36]<br>36% [37]<br>29% (Indian), 25% (Pakistani), 27% (Bangladeshi) [39]<br>88.33% [38]<br>34.1% 16-23 weeks, 58.7% 24-31 weeks [40]
Delayed (7+ months/27+ weeks)	13% [34]<br>15% [37]<br>1% (Indian), 2% (Pakistani), 1% (Bangladeshi) [39]<br>10.55% [38]<br>58.7% 24-31 weeks, 2.9% ≥32 weeks [40]
